# Characterization of the Core Determinants of Social Influence From a Computational and Cognitive Perspective

**DOI:** 10.3389/fpsyt.2022.846535

**Published:** 2022-04-18

**Authors:** Hyeji Lee, Dongil Chung

**Affiliations:** ^1^Department of Biomedical Engineering, Ulsan National Institute of Science and Technology (UNIST), Ulsan, South Korea; ^2^Department of Psychology, The University of Edinburgh, Edinburgh, United Kingdom

**Keywords:** social influence, computational modeling, individual differences, computational psychiatry, context dependence

## Abstract

Most human decisions are made among social others, and in what social context the choices are made is known to influence individuals' decisions. Social influence has been noted as an important factor that may nudge individuals to take more risks (e.g., initiation of substance use), but ironically also help individuals to take safer actions (e.g., successful abstinence). Such bi-directional impacts of social influence hint at the complexity of social information processing. Here, we first review the recent computational approaches that shed light on neural and behavioral mechanisms underlying social influence following basic computations involved in decision-making: valuation, action selection, and learning. We next review the studies on social influence from various fields including neuroeconomics, developmental psychology, social psychology, and cognitive neuroscience, and highlight three dimensions of determinants—who are the recipients, how the social contexts are presented, and to what domains and processes of decisions the influence is applied—that modulate the extent to which individuals are influenced by others. Throughout the review, we also introduce the brain regions that were suggested as neural instantiations of social influence from a large body of functional neuroimaging studies. Finally, we outline the remaining questions to be addressed in the translational application of computational and cognitive theories of social influence to psychopathology and health.

## Introduction

Most human decisions are made among social others. It is broadly observed that individuals' choice patterns sometimes vary and reflect the social information (1, 2). These phenomena highlight the importance of the social context at which the decision-making is taking place. Individuals being exposed to such “social influence” may have positive consequences; the decision maker whose actions were swayed by observing others' choices may benefit from the influence (e.g., joining others in following daily athletic routine) or get oneself to participate in spreading the good deed (e.g., ALS Ice bucket Challenge). However, in many other occasions, social influence is considered as a crucial factor that affects individuals negatively. For example, negative peer influence is known as a major risk factor for early initiation of substance use and other risky behaviors (3), and in line with this, having close friends and family members who suffer from substance use disorder is one of the prominent predictors for individuals' substance use problem (4). These bi-directional impacts of social influence suggest that the mechanisms how social information affects individuals could be quite complex.

There has been abundant amount of research carried out to understand the breadth and levels of social influence in individuals' choices. In classic social psychology studies, researchers largely focused on the impact of social environment in adolescents, given that adolescence is a critical neurodevelopmental period (5, 6). Due to the complex nature of the natural settings, analyzing questionnaire data based on self-reports was not sufficient to answer why the impacts of social environment on adolescents' delinquent behavior sometimes are positive (7) but some other times negative (8, 9). Addressing this issue, over the recent two decades, various types of experimental paradigms have been suggested to examine the impact of overt (e.g., advice from an expert) (10, 11) and covert (e.g., presence of peers) (12) social contexts. In parallel, computational modeling of behavioral data from laboratory settings has been found useful in disentangling potential factors and plausible neurobehavioral mechanisms underlying social influence. Yet, experimental designs in laboratory settings are typically restricted by the specific factors-of-interest (e.g., age group, delivery methods or contents of social information) in line with their hypotheses, and thus suggested computational models still have room for improvement.

In this review, we aim to review previous research on social influence from various fields of studies, and to suggest core factors that would play key roles determining how individuals process and respond to social contexts. In the next section, we overview the recent computational approaches suggested to explain why and how individuals are affected by social contexts. In the following three sections, we review three dimensions of determinants that are known (or expected) to modulate the extent to which individuals are influenced by others: characteristics of the individuals who are receiving the social influence, the forms that the influence is conveyed, and the domains and processes of decisions that the influence is modulating. In the last section, we discuss about future directions in understanding of social influence and its translational application to mental illness. Large proportion of the studies we include here also provided functional neuroimaging results, which further supported their suggested cognitive and computational models explaining how social information is involved in decision processes. Thus, whenever found necessary, throughout the current review, we also introduce the brain regions that were suggested as neural instantiation of social influence.

## Computational Models of Social Influence

How does an individual make decisions under social influence? To answer this question, we need a better approach than simply observing individuals' behavioral patterns, because there could be different paths of decision processes that underlie the same exact choice. To shed light on the question, various studies in social influence used computational modeling approaches in conjunction with functional neuroimaging (13–20). Given that social information contributes to change of individuals' initial decisions, the extent to which individuals use or respond to social information is often explained within the framework of learning. However, depending on the specific goal of the task and the way how the social information is framed, potential motives that individuals are expected to show differ (e.g., following the norm, or collecting more information) and moreover, different learning models are suggested to best explain individuals' choice patterns (e.g., Rescorla-Wagner type reinforcement learning model, or Bayesian learner model) [for review, see (21, 22)]. In this section, we review putative mechanisms of social influence suggested in these recent studies following basic levels of computations involved in decision-making (23): valuation, action selection, and learning. Of note, we focus on cognitive processes that occur within individuals who are on the receiving end of the social information, and the mechanisms how one may decide to exert influence over others [e.g., (24)] or how social information diffuses over a large group of people [e.g., (25)] are out of the scope of the current review.

### Adjustment of Individuals' Preferences

Under social context, on average, people tend to follow others' choices [(2); c.f., (26)]. One of the simplest explanation why people follow others' choices is that individuals become similar to social others who they are with. Previous studies suggested that having chances to observe others' choices sways individuals to change their own preferences—behavioral tendency how they make choices (*action selection*) in a particular context—to match that of social others. Individuals showed shifts in the extent to which they discount delayed rewards after observing the choices of the majority of the social group (27). Such a “contagion” of preference was observed even in the case when individuals were presented with choices from anonymous few social others rather than from a representative group. Individuals changed their choice behaviors (e.g., delayed reward, uncertain gambles, moral choices) after participating in a task phase where they were asked to predict others' choices, and the changes were explained by computational models that assumed shifts in individuals preferences toward the observed social others (19, 28, 29). These modeling results were corroborated by model-based neuroimaging results. Specifically, event-related blood oxygen-level-dependent (BOLD) responses in the dorsomedial prefrontal cortex (dmPFC), a brain region known to be recruited for social information processing (30–32), tracked individuals' beliefs about others' choices (19, 27). This set of results suggested that individuals adjust their preferences in the direction that matches with social others, and in turn, show conforming behaviors.

### Social Valuation

As any other decisions individuals make in life, choices under social contexts can be attributed to individuals' *subjective valuation* (33). This view assumes that individuals place value on the information obtained from social others and this additional social value can explain why they tend to make the same choices as social others. In contrast to the studies that reported individuals' preference change under social context, task contexts where individuals had a brief chance to observe others' choices successfully showed evidence for a transient use of social information. In recent studies, Chung et al. (17, 20) used a formal model comparison and showed that a brief observation of social others' choices may affect individuals in their valuation rather than changing their preferences; the impact of observing others' choices on valuation was defined as “other-conferred utility”. Consistent with their model-based results, it was observed that the ventromedial prefrontal cortex (vmPFC), a brain region known to encode subjective values of social and non-social choices (13, 34, 35), tracked trial-by-trial decision values combining the social values in individuals' decision processes (17). Such an impact of social valuation was also observed in a learning context where individuals made choices whether or not to follow social others' advice (15). Specifically, individuals' advice following behavior was explained by their adaptive learning process in which the value of obtained reward (or punishment) gets modulated for the choices advised by others. This value level premium, termed as “outcome-bonus”, was tracked in the septal area and the caudate, brain regions implicated in signaling rewards and reward prediction errors (36–38). Another recent study suggested that individuals may encode social value in the anterior cingulate cortex (ACC) through vicarious simulation conducted from observing others' choices, and that this distinct value signal is combined with experience-based value signal in the vmPFC for subsequent decision-making (39). These results suggest that individuals' motivations to conform emerge from their computations of the value of social information and/or the value of sharing membership with the social group.

### Learning From Social Others

The two perspectives introduced above are not mutually exclusive, but rather intertwined one another (22, 23). At a first look, the results would seem contradictory such that some studies suggest stable and non-changing individual preferences [e.g., (17)] whereas others suggest changes in preferences under social context [e.g., (19)]. However, social *learning* framework provided explanation why and how such subtle differences in the contexts may trigger differential responses from individuals. When individuals receive social information that is deviant from their own, BOLD responses in the dorsomedial prefrontal cortex (dmPFC) associated with social and cognitive conflicts were observed (14, 40, 41). Moreover, it was shown that this error signal is used as social prediction error, which individuals use to reduce the difference between self and others by learning from social others (14, 40, 42, 43). When individuals do not have a full access to social others' choice preferences or intentions (as in most of the social interactions), but believe others' choices are informative, individuals have to infer what others would be thinking to optimize one's own actions. In these contexts, individuals make inference about reliability of others' choices (44, 45), emulate others' intention (46), and combine the inferred social information with their own (44–47). This set of results suggests that individuals are influenced by social contexts because they use the information in learning how to adjust their choices at a specific context (e.g., interacting with the same social partner repetitively, observing choices of randomly assigned partner).

### Summary

As briefly reviewed above, cognitive mechanisms of social influence may take different forms depending on the context in which its impact is examined. Depending on how the social information is provided, individuals may use the information as a transient nudge toward others' opinion or as a normative guide directing them to be changed. To date, computational modeling approach has been found useful in delineating such variant mechanisms (21, 22, 48, 49). However, there are still many remaining questions regarding the mechanisms, such as why some individuals are more susceptible to social information, and how does the value of a certain type of social information determined. To address these, we suggested that further practices in quantifying potential modulatory effects of latent variables are crucial. In the following sections, we review studies on social influence from various fields of studies and highlight three dimensions of determinants that are known to modulate the extent to which individuals are influenced by others.

## Cognitive, Psychological, and Contextual Determinants of the Impacts of Social Influence

### Who Is More Susceptible to Social Influence

Everyone is bound to live under social influence, but some are more affected by others. Over the decades, a considerable amount of literature in social psychology has been published on the association between individual-specific characteristics and the extent to which individuals are influenced by social influence [e.g., (50, 51)]. The individual-specific characteristics that have been investigated across various fields of studies include demographics (e.g., age, socioeconomic status) and individuals' psychological characteristics (e.g., anxiety level, self-esteem). In this section, we review major factors that may mediate or modulate the impact of social influence on individuals' decision processes.

#### Demographic Factors: Age and Socioeconomic Status

Age has been considered as one of the most salient determinants that modulate social influence. Early pioneering research focused on the negative impacts of peers on adolescents' behavior. A seminal work by Gardner and Steinberg (10) showed that adolescents, compared to adults and young adults, take more risks when in peer groups. Adolescents' increased risk-seeking behavior was accounted for by the imbalance between adolescents' reward and cognitive control circuits (52, 53). In line with this neurodevelopmental model, their heightened social susceptibility was suggested to be associated with socio-emotional neural system (54, 55). Supporting these neural sensitivity models for adolescents, adolescents who exhibited increased risk-taking choices under the presence of peers indeed showed increased BOLD responses in the reward circuit, including the ventral striatum and orbitofrontal cortex (12).

In contrast to classic studies on social influence in adolescents, recent studies gave more attention to positive impacts of social influence (56). Do et al. (57) specifically compared adolescents' conforming behaviors toward different types of social influence. In this study, adolescents tended to stick to their original attitudes toward various types of behaviors, but on the cases when they change their attitudes, adolescents conformed to constructive behaviors (e.g., working hard in school) more than unconstructive behaviors (e.g., smoking a cigarette). Another study used computational modeling approach and showed neural and behavioral evidence for positive peer influence in adolescents (20). Adolescents were making a series of gamble choices and presented with social others' choices before they made each choice. Consistent with the results observed in adults (17), adolescents followed others' choices on average, and such conformity was explained by added social value to the option others chose. In particular, adolescents who never used any types of substances were influenced by others' safe choices, whereas adolescents who have used were not. Although these studies did not directly compare adolescents' decision patterns from those of adults, the results suggested the mechanisms how individuals use social information in their adolescence, a sensitive period for sociocultural processing (6).

Considering the hormonal effects on biological development of the brain, one should consider pubertal stage as a determinant as important as age in developmental research. Indeed, across many adolescent studies, it has been reported that the extent to which individuals are susceptible to social influence is heightened during adolescence and usually diminished after pubertal growth (55, 58, 59). Moreover, recent functional neuroimaging studies suggested that puberty might play a more important role than chronical age in structural and functional development of the brain [(60); for review, see (61)]. This set of studies again highlights that individuals' age would explain considerable variability in their neural and behavioral patterns reflecting individual differences in social information processing.

Another noteworthy demographic factor is socioeconomic status (SES). There have been fairly consistent results suggesting that individuals' socioeconomic status has a significant effect on their behavior in social context. Psychological research suggested the association between individuals' social class and their perspectives over the social environmental (62). Specifically, individuals' high and low classes were considered to be shaped by abundance (or scarcity) of available resources, which in turn may underlie their behavioral tendencies either to focus on one's own internal states or to external factors (62). Consistent with this view, empirical research on social influence among marginalized groups also reported that they tend to conform to their peers more not to be excluded from their community and assert their identity in the group (63).

Recent neuroimaging research further supported the role of SES in individuals susceptibility to social influence. Casio et al. (64) examined whether individuals' SES moderates the relationship between brain responses to social exclusion and the extent to which they conform to peer influence. Specifically, individuals who had low SES showed positive association between neural sensitivity to social exclusion measured in the “social pain” network regions [including dorsal anterior cingulate cortex (dACC), anterior insula, and subgenual cingulate cortex (subACC)] and their conforming tendencies, whereas individuals who had high SES showed the opposite association. Comparable moderating effects of SES were observed for the brain regions implicated in mentalizing [e.g., medial prefrontal cortex (mPFC), temporoparietal junction (TPJ)] (64, 65). These results together imply that SES is neurocognitively linked to the way people process social information.

Of note, the measurements of SES vary across studies and these results should be interpreted with caution. The most common indices include income and educational levels (64, 66), and subjective assessments, such as perceived neighborhood quality (67) and the MacArthur ladder, which measures individual belief about one's location in a status order (65). Although these assessments are usually correlated, they should not be used interchangeably, because they might have enough differential effects on the brain development (68).

#### Psychological Characteristics: Anxiety and Self-Esteem

Among individuals who have the same demographic profiles, social influence still may have very different impacts, contingent upon individuals' psychological characteristics. Given the social characteristic of the information processing, social anxiety is one of the closest psychological factors that may modulate the effect of social influence. A recent study reported that individuals' social anxiety was positively associated with their conformity to bullying under social influence, such that individuals who show highest social anxiety level conforms to others the most (69). Even in learning directly from experience, highly anxious individuals showed a negative bias (i.e., learning better from bad news) when social others were observing (70). Another study examined social influence differences between healthy individuals and individuals with social anxiety disorder (71). Consistent with the results from the subclinical population, individuals with social anxiety disorder showed higher susceptibility to social influence particularly when social others rated presented face as more attractive than they originally reported. This result was interpreted as evidence for increased motivation to pursue social acceptance and avoid social rejecting in individuals with high social anxiety.

Self-esteem is another psychological characteristic that may be associated with the extent to which one is swayed by others' opinion. Indeed, various classic social psychology research have examined whether individuals' self-esteem is a major moderator of social influence (72–74). Despite the general results showcasing negative association with individuals' susceptibility to peer influence—individuals with low self-esteem are more susceptible to others' influence (72, 74)—, other studies suggested that the relationship is rather more complex. Nisbett and Gordon (73) suggested that modulating effect of self-esteem may differ depding on the type of social influence. Particularly, individuals' self-esteem was negatively associated with the extent to which they are influenced by others for the type of social influence that is relatively easy to comprehend but implausible, while the association was non-monotonic or even opposite for a difficult but plausible message.

Recent neuroimaging studies corroborated this suggested association between self-esteem and their susceptibility to social information. Somerville et al. (75) reported that individuals who had low self-esteem not only reported that they received positive feedbacks less from others, but also were more sensitive to positive feedbacks received by others compared with individuals who had high self-esteem. This result implied that social feedbacks might be exaggerated in low self-esteem individuals, and thus have increased susceptibility to social influence. Will and colleagues (76) used computational modeling approach and suggested that individuals' self-esteem is established through the way how they learn about social others. These results altogether hint a possibility that self-esteem is more than a modulator for individuals' social susceptibility, but rather a dynamically changing characteristic shaped by the history of social interactions.

#### Summary

We reviewed various individual characteristics that are associated with the extent to which individuals are influenced by social contexts. As introduced above, vast amount of studies showed that a large variance of individual differences exists in susceptibility to social influence. However, only few studies directly took these associated factors into account in constructing a cohesive computational model of social influence. Individual characteristics such as age and socioeconomic status may be closely tied to developmental changes or differential learning experiences, while other characteristics (e.g., anxiety and self-esteem) may be linked to baseline traits each individual has and to a specific state individuals reside at the moment. Better mechanistic understanding of social influence spanning across these individual characteristics may provide explanation why minorities who are most vulnerable (e.g., adolescents), or marginalized and stigmatized cohorts are more susceptible to their social environment (3, 12) and even likely to experience mental health problems (77, 78).

### How Is the Social Influence Conveyed

Sometimes what matters is how you say it, rather than what you say. In the same vein, the exact same content can have a very different impact on people's behavioral changes depending on from whom or how it is delivered. Characteristics of the group (e.g., social distance, expertise) may shape the credibility of the social information, and thus individuals may be more (or less) influenced by a particular social group. Two distinctive ways of being exposed to social information includes directly observing others and in reverse, realizing that one is being observed by others. Depends on these specific circumstances, individuals may obtain different types of social information and in turn, be influenced differently. In this section, we review previous research that examined how the forms of social influence modulate the way how or the extent to which social influence affects individuals' choices.

#### Characteristics of Others: Social Closeness, Credibility, and Competence

When one has a chance to decide on the team members to work together, one would usually prefer others who he or she shares similar perspectives and relates one another easily. A biased behavioral tendency of being assorted based on individuals' preference is often observed in social context, such that individuals who are closer in their social network are more likely to have similar preference (79). Moreover, social closeness, a psychological construct that is well-described as a shared variance between oneself and others (80), was shown to have a significant effect on individuals' judgement about others (81). In other psychology studies where a dichotomous classification of social relationship is adopted (in- vs. out- group) showed consistent results, such that individuals showed a biased preference toward in-group members (82). Such biases toward socially intimate others might be accounted for by their motivation to keep their membership stable and to enhance self-esteem (2).

Recent neuroimaging studies presented further evidence explaining why and how such biases exist. Sip et al. (83) examined whether social feedbacks from a gender-matched close friend vs. from a confederate have differential impacts on individuals' decision pattern and on their neural responses. Individuals were responsive to social feedback and showed changes in choice patterns accordingly, but only when the feedback came from a close friend. This effect was reflected in BOLD responses in the vmPFC and posterior cingulate cortex (PCC), which they presented as supporting evidence for modulatory impact of social closeness on decision-making processes. A similar study that examined individuals' neural responses to social influence revealed differences when the influence originated from in- vs. out- group (84). Particularly, a set of brain regions including the medial prefrontal cortex (mPFC), amygdala, and ventral striatum (vStr) showed higher BOLD responses for the social influence from in-group than out-group members. Consistent with these findings, the default mode network (85), a set of brain regions including the medial PFC and PCC, and its interaction with subcortical regions are known to be closely associated with mental representation about self-other relationship (86). These studies together highlight that social closeness is an important determinant for social information processing.

Another very closely related factor is whether the achieved social information is perceived useful or not. When expertise of social others is explicitly informed, one can use this knowledge to judge whether social information from them is reliable or not. Supporting this view, various studies have shown that people tend to follow opinion and advice from people with expertise than from novice (87, 88). Klucharev et al. (89) suggested that presenting an object paired with an expert enhances memory performance and moreover has a positive impact on the attitude toward the object. Such an impact of perceived expertise was associated with re-evaluation of an item (89, 90), which may account for the reason why people are more likely to follow experts' opinion.

It is important to note that in most of the cases, it is not obvious whether the social information is useful or not. Thus, individuals should estimate how useful the social information is to maximize one's own benefit (or minimize the harm). As crude heuristics, opinion from larger group of people can be taken into account more heavily (45), and others' faster responses are considered more informative (91). Independent of the true usefulness of the information, individuals were more likely to be persuaded by others when presented with higher confidence (92, 93). Evaluation of the confidence that is presented for (or estimated to be associated with) the social information was tracked in the vmPFC, an area dissociable from the region that encoded subjective value signal combining one's own and others' preference (44, 94). These results support the view that by estimating who knows better or whether the social information is useful, individuals can choose their strategy to learn from social others (13, 95).

#### The Way Social Information Is Given: Observing and Being Observed

When being around social others, there are different ways to acquire additional social information. The type of information one can achieve is yoked to the methods how social influence is acquired, and thus how one processes and uses the information naturally should be different accordingly. The most direct way to acquire social information is through a chance to observe others' choices which inform others' preferences and social norms. Chung et al. (17) showed that individuals tend to follow others' choices during risky decision-making. By conducting a formal model comparison, they suggested that such conformity is explained by a value-based decision process combining additional utility to the option chosen by others, rather than by changing individuals' original preferences. The mechanisms how individuals combine their own knowledge and preference with social information may vary. Individuals may project their own preference in predicting that of others (96), and also track whether others' intentions underlying the observed actions of others change over time (97). Other studies suggested that individuals use social information to adjust their own opinion and intend to match with that of others. Specifically, when individuals were asked to report attractiveness of a series of faces after viewing others' responses, their original attractiveness reports were adjusted toward the others (40, 98). These results suggested that individuals are able to track the difference of the values (or preference) between their own and others (17, 40, 41), and change their choices (or ratings) accordingly to minimize the difference (40).

On other occasions, one can be mindful of being around others, but have no chance to directly observe others' choices. The impact of simple presence of others is largely investigated in adolescents, where presence of friends were found to increase adolescents' risk seeking behavior (10, 12, 99). Individuals tended to show higher sensitivity to rewards and more impulsive choices under presence of others even if the social others were not friends, but strangers (100). Such social influence was attributed to social reward, associated with approval from others (101). In a recent study, Powers et al. (102) also examined impacts of the contexts where friends were simply present at the same room or monitoring participants' choices. Particularly, options were more likely to be chosen when they were paired with friends' monetary gains compared with when they were paired with friends' losses. In adults, such adjustment of individuals' choice attitudes were more pronounced when friends were monitoring the choices than merely present, while adolescents showed comparable responses to the social contexts regardless of whether friends could witness the choices or not. These results suggested that individuals may take into account wellbeing of friends, particularly when others can immediately witness the choices.

Individuals may infer what others would expect from their choices and place social values toward meeting the inferred expectation (13, 46). This perspective was closely examined in a recent study where participants were asked to predict others' choices (19). After successfully learning others' choices, individuals' preferences for risky choices changed toward that of others as if there was a “behavioral contagion”. The main goal of predicting others' choices might have motivated individuals to simulate others' preferences and mentalize (103), which may underlie why social context affects individuals differently.

#### Summary

We reviewed that how social influence is conveyed may shape the mechanism how a social context would affect individuals' choices. When individuals are under a social context, they may start extracting a set of information ranging from whether others share the same goal as them to whether others have more amount of information. In the inference process figuring out social others' goals, individuals may recalibrate their subgoals [e.g., to collaborate or compete with others, to mimic others' actions (104), to meet a consensus (105)]. Given that real world is largely uncertain and volatile, we, as social agents, must be constantly solving such an inference problem to first evaluate the usefulness of social information and next alternate how to use the information (46).

### What Decision Domains and Processes Is the Social Influence Applied to

Would a person who is susceptible to one type of social information always be sensitive to other types of social contexts? It is not uncommon in real life that the extent to which individuals respond to social information differs depending on the type of behavioral choices which are subject to the influence. For example, an adolescent who is not swayed by aberrant behaviors of peers may show tendencies to join her friend for volunteer opportunities, and an addict who easily gives in to craving around other substance users may not respond to intervention of social support groups. In this section, we review previous studies in social influence across different decision domains and processes. In addition, we discuss whether or not social influence is domain-general and if not, whether there are any latent variables that explain why individuals show domain-specific responses to social information.

#### Domain-Specific and Domain-General Mechanisms of Social Influence

Social influence can be readily observed in almost every kind of decision process in our life. Mirroring this, there were many empirical studies ranging from the simplest perceptual decision-making to complex moral decision-making where they used a variety of task paradigms to show the effects of social influence on human information processing. Perceptual decision-making tasks are based on the evaluation of sensory information, such as the length of lines (106), the dominant color of the presented patches (107), or the shape of three-dimensional objects (108). Personal preference tasks include variety of options, such as preference for t-shirts (41), faces (40, 98), and works of art (108, 109). In monetary reward tasks, there are explicit gains and losses of money associated with each of the choice option (17, 19, 110). Lastly, in social preference tasks, individuals encounter decision-making situation where they have to consider explicit losses and gains of social others and their own simultaneously (29, 101). On average, behavioral changes indeed were observed under social influence across all of these studies that probe different levels of cognitive processing in humans. However, due to the variety of contexts each study adopted (e.g., cover stories) and the differences in the targeted cognitive processes (e.g., perception, valuation), there is no universal computational framework that explains the mechanism of social influence across domains.

There are a few computational frameworks that provided cross-domain accounts for social influence (21, 22). First, individuals may be trying to learn others' preferences and values under social influence. Such “normative influence” of social contexts, where social others' choices are not necessarily based on a better set of information, were explained by reinforcement learning (RL) framework capturing individuals' change of behaviors toward others [(40, 98), c.f., (111)]. Consistent with this perspective, individuals under social context were sensitive to the opinion differences between them and the others (41, 112), and it was shown that a set of brain regions involved in social and monetary reward learning overlap (113, 114). The RL framework successfully captured the extent to which individuals conform to others' preference-based choices over primary and social rewards (40, 115). Second, individuals may be collecting more information from others' choices. Following such “informational influence” of social contexts, individuals seemed to be using others' responses and choices to appropriately adjust their original responses. To integrate information from two sources, individuals computed the importance and reliability of each piece of information (44, 45, 47). Such a Bayesian learner framework successfully explained individuals' conformity not only in perceptual, but also in value-based decision-making particularly when statistical inference was available.

It is worth noting that behavioral patterns which are well-explained by the same computational framework may in fact induced by different neural mechanisms; differential implementation level explanation as per Marr's three levels of analysis (116). For example, a recent study directly compared multivoxel neural patterns for social conformity with that for classic reward learning, and suggested that neural responses in the brain regions typically involved in non-social RL (e.g., striatum) do not explain whether or not individuals conform to social information (111). This emphasizes again the importance of interdisciplinary approaches in understanding human information processing. A careful consideration of specific contexts will shape individuals' motivation (“computation level”), but why and how individuals process social information in the context need thorough examination not only in algorithmic level (e.g., computational modeling) but also in implementational level (e.g., functional neuroimging) (117).

#### Summary

We briefly reviewed plausible mechanisms suggested to date of social influence over different decision domains. Although cognitive motivations defined over psychological constructs including value and information maximization accounted for neural and behavioral mechanisms under social influence (33), applying the same mechanism to different levels of cognitive processing has been challenging, because task settings (e.g., goal, order, amount of information) also varied across different studies. Future studies may tailor the study design to specifically examine individuals' cross-domain susceptibility to social information. By using the same task settings, but over different domain, we would get a direct chance to address whether individuals' domain-specific sensitivity and confidence, which will be manifested as preference for social information, affect the extent to which individuals use social information.

## Concluding Remarks and Future Challenges

Research on social influence has been conducted across various fields of studies. Recent computational approach in conjunction with functional brain imaging technology provided new impetus for the study of social influence, and shed light on underlying mechanisms of individual cognitive processes under social context. Still, there are major challenges remaining given the sheer diversity of social contexts. In this review, we overviewed previous studies in social influence along the three axes of determinants (who are the receivers, how is the influence provided, and to what is the influence applied) that may modulate and mediate the impacts of social influence. These three dimensions are not mutually exclusive one another and thus, they would not completely compartmentalize the impacts of one axis from the other. Still, we hope that our review would highlight potential co-factors crucial to consider for expanding our mechanistic understanding of social influence to translational applications (e.g., intervention design) (118, 119).

Given the complex nature of social contexts, simply adding up all the plausible factors into one experiment might not bring solutions. To address this issue, coherent and theory-driven computational modeling approaches should be proceeded (22, 120, 121). In parallel with this formal theory-driven approach, individual differences and extreme cases (e.g., cultural differences, race and gender discrimination, mental illness) cannot be overlooked as described herein. Thus, hypothesis testing in special population may provide further insights in examining the generalizability and transferability of the model (122–125). As an equally important research direction, data-driven understanding of behaviors in social contexts may provide complementary insights for latent variables. Nowadays, taking advantage of large-scale studies and open science practices, we now have better access to big data including personal habits and their social network (126–128). However, we still have to interpret the results with caution considering the sparsity and multi-dimensionality of the data (129).

Considering the importance of both theory-driven and data-driven approaches in mind, there are at least two issues to take into account when in designing future studies in social influence. First, dimensional measurements of potential determinants are preferrable than to have dichotomized classes. For example, most of the studies that investigated the impact of social closeness took contrast approach where the effects of a close friend vs. a stranger were examined (82–84). However, social closeness is not only associated with perception of social membership, but also trust and competence (130, 131). That is, we cannot disentangle potential effects of social distance and other co-varying factors by having only two categories along the dimension. Second, volatility of the social context should be considered to better mimic real world interactions. Social environment and relationship between people constantly change and how we perceive the context gets adjusted accordingly. In perspective of formalizing its impacts in the model, changes in belief about others' advice (13) or active alterations between utilized strategies for social information (46) can be implemented. Alternatively, to explore naturally emerging dynamics in rich environment, new experimental designs may target for collecting neural and behavioral data from interactions between uncontrolled real dyads simultaneously (132–134), and even further, using naturalistic social stimuli such as real-time videos and virtual reality (135). Using naturalistic social environment would get us closer to directly simulate the impacts of social contexts simulating translational applications. However, as reviewed above, there are numerous factors that are already known to affect social processing, but we have close to no understanding how these factors interact and interfere each other. Thus, for broader generalizability and future individualized translational applications, it cannot be emphasized enough the importance of compartmentalized and computational understanding about the underlying determinants of social influence.

## Author Contributions

HL and DC conceptualized the study, wrote the first draft of the manuscript, and contributed to manuscript revision, read, and approved the submitted version. All authors contributed to the article and approved the submitted version.

## Funding

DC was supported by UNIST internal funding (1.220032.01) and the National Research Foundation of Korea (NRF-2020S1A3A2A02097375). The funders had no role in study design, decision to publish, or preparation of the manuscript.

## Conflict of Interest

The authors declare that the research was conducted in the absence of any commercial or financial relationships that could be construed as a potential conflict of interest.

## Publisher's Note

All claims expressed in this article are solely those of the authors and do not necessarily represent those of their affiliated organizations, or those of the publisher, the editors and the reviewers. Any product that may be evaluated in this article, or claim that may be made by its manufacturer, is not guaranteed or endorsed by the publisher.
